# The utility of a pleural carcinomatosis score for assessing resectability

**DOI:** 10.1515/pp-2025-0033

**Published:** 2025-12-16

**Authors:** Gabrielle Drevet, Yaniss Belaroussi, Valentin Soldea, Erik Kovacs, Renaud Grima, Jean-Michel Maury, François Tronc

**Affiliations:** Department of Thoracic Surgery, Lung and Heart-Lung Transplantation, Hôpital Louis Pradel, Hospices Civils de Lyon, Lyon, France; EA 3738 CICLY, Université Claude Bernard Lyon 1, Lyon, France

**Keywords:** malignant pleural effusion, videothoracoscopy, pleural carcinomatosis, score

## Abstract

**Objectives:**

Malignant pleural effusion is a common evolution of various cancers and is associated with poor prognosis and quality of life. Currently, the surgical approach is mainly palliative, involving videothoracoscopic talc pleurodesis or the insertion of indwelling pleural catheters. No surgical options with a curative intent are validated for this indication. The aim of our study was to evaluate a score for assessing resectability in pleural carcinomatosis.

**Methods:**

122 consecutive patients with recurrent symptomatic pleural effusion, referred to our thoracic surgery department for a surgical exploration of the pleura, were prospectively included. Each patient underwent a detailed description of videothoracoscopic findings, which were summarized in our pleural carcinomatosis score. Resectability was then discussed in our surgical staff meeting.

**Results:**

Eighty patients (65.6 %) were diagnosed with metastatic pleural spread, while 42 patients were diagnosed with benign pleural disease (34.4 %). Patients diagnosed with benign pleural effusion had a median score of 2, and 29 patients (69 %) were considered resectable. Patients diagnosed with malignant pleural effusion had a median score of 11, and only 13 patients (16.2 %) were considered resectable. Those deemed resectable had a median score of 5. The threshold for resectability in our score was set at 6.

**Conclusions:**

The meticulous exploration of the pleura and calculation of the pleural carcinomatosis score could aid in selecting patients for curative-intent surgery. Patients with a score equal to or less than 6 should be discussed in a multidisciplinary tumor board where the possibility of surgery is considered.

## Introduction

Pleural disease affects over 3,000 people per million population each year worldwide [1]. While medical history, physical examination, and pleural fluid cytology enable a diagnosis in nearly half of cases [2], the other half of patients will need further explorations. In patients with a good performance status, thoracoscopy is recommended [3], allowing simultaneous pleural effusion drainage, pleural exploration, pleural biopsy and pleurodesis. In case of a malignant pleural effusion, the prognosis is poor, with median survivals ranging from 3 to 12 months, depending on the stage and type of the underlying malignancy [4]. Malignant pleural effusion is a common manifestation of various cancers, predominately lung cancer in men and breast cancer in women [5]. The current therapeutic approach is primarily palliative, involving videothoracoscopic talc pleurodesis or the insertion of indwelling pleural catheters, possibly associated with systemic therapies [6]. The main goal of surgery in this indication is to alleviate symptoms and prevent pleural fluid re-accumulation. In these cases, therapeutic strategy is often protocol-driven, regardless of pleural tumor burden. Currently, no specific evaluation of the pleura is validated, and all patients are typically offered palliative surgery. There are no recommended curative-intent surgical options in this indication, but there is a need for advances to improve evaluation and offer specific treatments for patients with malignant pleural effusions. The aim of our study was to evaluate the accuracy of a pleural carcinomatosis score and assess its utility in determining resectability.

## Materials and methods

### Study design

This observational study was conducted in the thoracic surgery department of the Louis Pradel Hospital (Lyon, France), department with expertise in the surgical management of neoplastic lesions of the pleura, and included prospectively all consecutive patients referred to our thoracic surgery department for recurrent pleural effusion between January 2020 and January 2021. Informed written consent was obtained from all eligible patients. The study was approved by The French Society of Cardio-Vascular Surgery (Société Française de Chirurgie Thoracique et CardioVasculaire, SFCTCV) and the Institutional Review Board number is IRB00012919. The study was designed following the Strengthening the Reporting of Observational Studies in Epidemiology (STROBE) guidelines.

- Population selection.

The inclusion criterion was the presence of a pleural effusion warranting surgical exploration. The non-inclusion criteria were: altered general condition (Performance Status greater than 2), contraindication to general anesthesia, patient’s refusal to undergo surgery, and/or to participate in the present study.

Collected data included: medical history of cancer, if applicable: primary tumor origin and presence or absence of extra-pleural metastases, pleural fluid cytology, date of surgery, score results, resectability according to the operating surgeon’s opinion, definitive pathological examination, postoperative complications and date of death or the most recent date the patient was confirmed alive.

b. Surgical procedure.

Videothoracoscopic exploration of the pleura was performed under general anesthesia, with a double-lumen bronchial tube inserted. The patient was positioned in lateral decubitus, and two 10-mm trocars were inserted into the chest wall: one in the seventh intercostal space in the mid-axillary line, the second in the fifth intercostal space in the anterior axillary line. The surgery was performed on excluded lung and a meticulous exploration was undertaken. A pleural tissue biopsy was then obtained, and surgical talc was instilled. A 24Fr chest tube was finally inserted and connected to a Pleur-Evac© system.

c. Pleural score construction.

**Figure 1:**
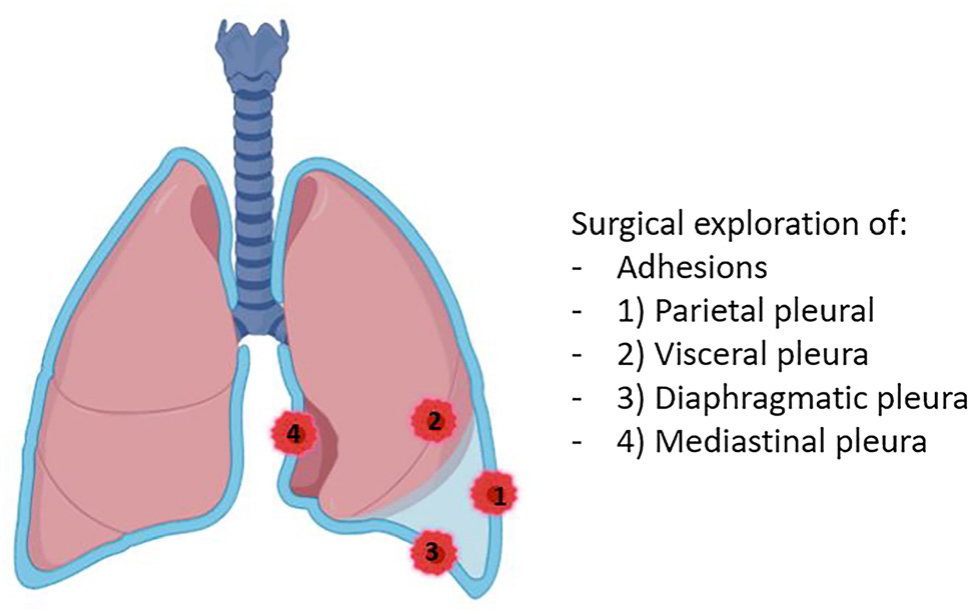
Surgical exploration of the pleura. Created with Biorender.com.

**Figure 2:**
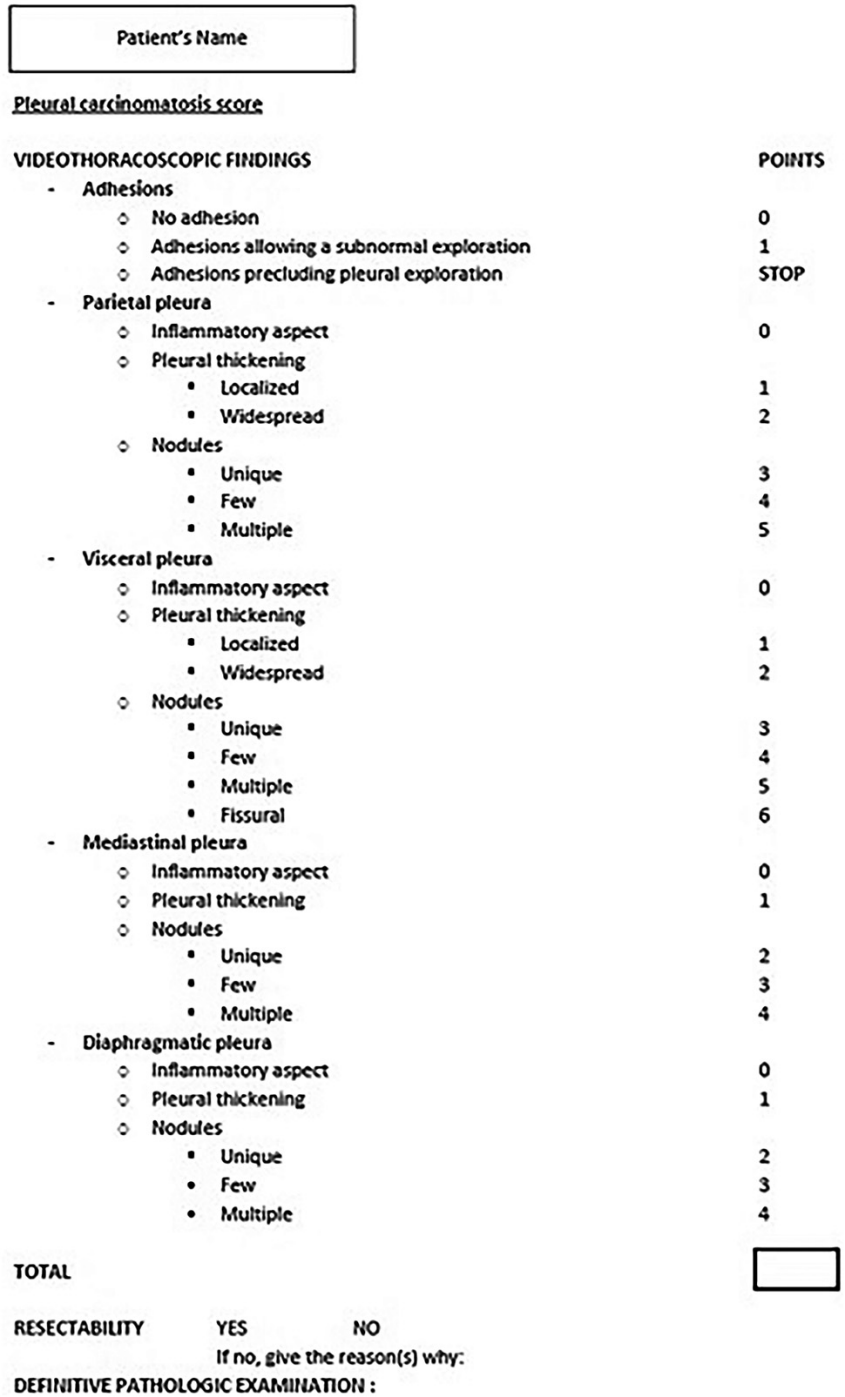
Pleural carcinomatosis score as it was used in the operative reports.

A data steering committee including, an oncologist, two surgeons and an epidemiologist gathered and developed a pleural score for resectability based on the physicians’ experience and the literature [7], [8], [9], [10]. Exploration of the pleura was divided into 4 compartments: the parietal pleura, the visceral pleura, the diaphragmatic pleura, and the mediastinal pleura (Figure 1). In each compartment, the pleura can be described as inflammatory, localized or widespread pleural thickening, presence of unique/few/multiple nodules. For the visceral pleura, an item has been added to note whether there are nodules in the fissure. Points have been allocated for each item, and all the points need to be added together to obtain the final score. A score from 0 to 20 can be obtained. The operating surgeon recorded a detailed description of videothoracoscopic findings in a dedicated report (Figure 2). Resectability was then determined by the operating surgeon and discussed in our surgical staff meeting.

### Statistical analysis

The primary endpoint was the association between the score and the determined resectability. Statistical analysis was performed using R Software, version 3.5.2. Quantitative variables were described by median and interquartile range (IQR) and qualitative variable by frequency and percentages.

We established a threshold for the resecability score. We determined the diagnostic performance of the score for resectability (sensitivity, specificity, positive predictive value, negative predictive value and their 95 % confidence intervals). Median survival was estimated from the date of surgery to the occurrence of death.

Based on the Bayes’ theorem and our results, a 10.7 % prevalence of resectability was used for the calculations. The performance was analyzed according to ROC curves related to it. We performed bootstrap calculation of the AUC within 1,000 re-samplings for validation.

## Results

A total of 122 patients underwent videothoracoscopic exploration of recurrent pleural effusion between January 2020 and January 2021. The median age was 70 years (IQR, 62 – 77 years). Fifty-five patients (45.8 %) had a medical history of cancer before surgery. Preoperative pleural fluid cytology was negative in 75 patients (61.5 %) (Table 1). As confirmed by surgical pleural biopsy, 80 patients (65.6 %) were diagnosed with metastatic pleural spread and 42 patients were diagnosed with benign pleural disease (34.4 %).

**Table 1:** Population’s characteristics.

	Overall n (%) or median [IQR]
**Clinical features**	
Sex	
Female	53 (43.4)
Male	69 (56.6)
Age	70.2 [61.9, 76.7]
Previous history of cancer	55 (45.1)
Lung cancer	12 (21.8)
Breast cancer	17 (30.9)
Prostate cancer	6 (10.9)
Other	20 (36.4)
Synchronus metastatis	29 (23.8)
Preoperative pleural fluid cytology	93 (76.2)
Negative	75 (61.5)
Positive with tumoral cells	34 (27.9)
Not performed	13 (10.6)
Surgical procedure	
Emergency surgery	40 (32.8)
Planned surgery	82 (67.2)
Pleural score calculated	104 (85.2)

Patients diagnosed with benign pleural effusion (n=42) had a median score of 2 (range=0 – 16). Four patients (9.5 %) had adhesions within the pleural cavity precluding an optimal pleural exploration. Twenty-nine patients (69 %) were considered resectable. The main diagnosis was nonspecific inflammation (29 patients, 69 %). Six patients (14.3 %) experienced post-operative complications (2 prolonged air leaks, 2 acute renal insufficiencies, 1 pneumonia and 1 hemothorax requiring surgical revision). Median survival for patients with benign effusion was 41.7 months (CI95 [30.4-not reached]).

Patients diagnosed with malignant pleural effusion (n=80) had a median score of 11 (range, 0–20). 12 patients (15 %) had adhesions within the pleural cavity precluding an optimal pleural exploration. The main diagnoses were lung cancer (34 patients, 42.5 %), pleural mesothelioma (18 patients, 22.5 %) and breast cancer (5 patients, 6.2 %) (Table 2). Twenty-three patients (28.7 %) experienced post-operative complications. Main complications were prolonged air leaks (6 patients; 7.5 %), pneumonia (4 patients; 5 %), atrial fibrillation (3 patients; 3.25 %) and recurrent pleural effusion (2 patients; 2.5 %). Post-operative death occurred in three patients (3.7 %); one patient suffered from post-operative cardiac tamponade and two patients developed acute respiratory distress syndrome. Median survival for patients with malignant pleural effusion was 15.9 months (CI95 [7.37–22.5]).

**Table 2:** The pleural carcinomatosis score with the number of patients presenting the different characteristics.

	Points	Overall n (%) or median [IQR]
**Pleural score**		104
**Adherences**		
No adherence	0	67 (64.4)
Adherences allowing surgical exploration	1	37 (35.6)
Major adherences making surgical exploration risky	STOP	18 (14.8)
**Parietal pleura**		4 [2, 5]
Inflammatory	0	18 (17.3)
Pleural thickness		
Localized	1	5 (4.8)
Generalized	2	20 (19.2)
Nodes		
Unique	3	1 (1.0)
Some	4	11 (10.6)
Multiples	5	49 (47.1)
**Visceral pleura**		1 [0, 4]
Inflammatory	0	44 (42.3)
Pleural thickness		
Localized	1	11 (10.6)
Generalized	2	17 (16.3)
Nodes		
Unique	3	0 (0.0)
Some	4	13 (12.5)
Multiples	5	13 (12.5)
Scissural	6	6 (5.8)
**Mediastinal pleura**		0 [0, 3]
Inflammatory	0	57 (54.8)
Pleural thickness	1	17 (16.0)
Nodes		
Unique	2	1 (1.0)
Some	3	13(12.5)
Multiples	4	16 (15.4)
**Diaphragmatic pleura**		1 [0, 3]
Inflammatory	0	45 (43.3)
Pleural thickness	1	24 (23.1)
Nodes		
Unique	2	2 (1.9)
Some	3	10 (9.6)
Multiples	4	23 (22.1)
		
**Total score**		8 [3, 13]
**Resectability**		
Resectable disease		
Not resectable		62 (52.5)
Resectable		42 (34.4)
If not resectable; reasons		
Major adherences		1 (1.6)
Diffuse damage		54 (87.1)
Major mediastinal disease		2 (3.2)
Undefined		5 (8.1)

In the subgroup of malignant pleural effusions, 13 patients (16.2 %) were considered resectable (Table 3). Patients deemed resectable had scores ranging from 0 to 12, with a median score of 5. Among them, two patients underwent resection. Post-operative courses were uneventful. Among the 11 patients considered resectable but not undergoing curative intent surgery, five patients presented with other metastatic sites, two patients died from post-operative acute respiratory distress syndrome, and two patients had significant coexistent medical condition rendering them unfit for aggressive surgical treatment.

**Table 3:** Tested thresholds.

Tested threshold	Sensitivity, %	Specificity, %	Positive predictive value, %	Negative predictive value, %
>5	83.9	81.0	86.9	79.1
>6	83.9	88.1	93.0	78.7
>7	79.0	90.5	92.4	74.5
>8	74.2	92.9	93.9	70.0
>9	64.5	97.6	97.6	65.0
>10	58.1	97.6	97.3	61.1

In terms of discriminatory power, the AUC (95 %CI) for the pleural score was 93.0 (88.4 – 97.6). When using bootstrap calculation for validation, the mean AUC was 92.9 (with a minimum AUC of 84.5, and a maximum AUC of 99.3). The threshold for pleural carcinomatosis resectability was determined to be 6, according to Younden’s index. Patients could benefit from curative intent surgery if their score was equal to or under 6. Patients with a score greater than 6 were mainly considered unresectable. Setting the threshold score at 5 (Table 3), the sensitivity was 83.9 % and the specificity was 80.1 % (All but one patient with a score greater than 5 had an unfavourable outcome. All but one patient with a score below 5 had a favourable outcome.

**Figure 3:**
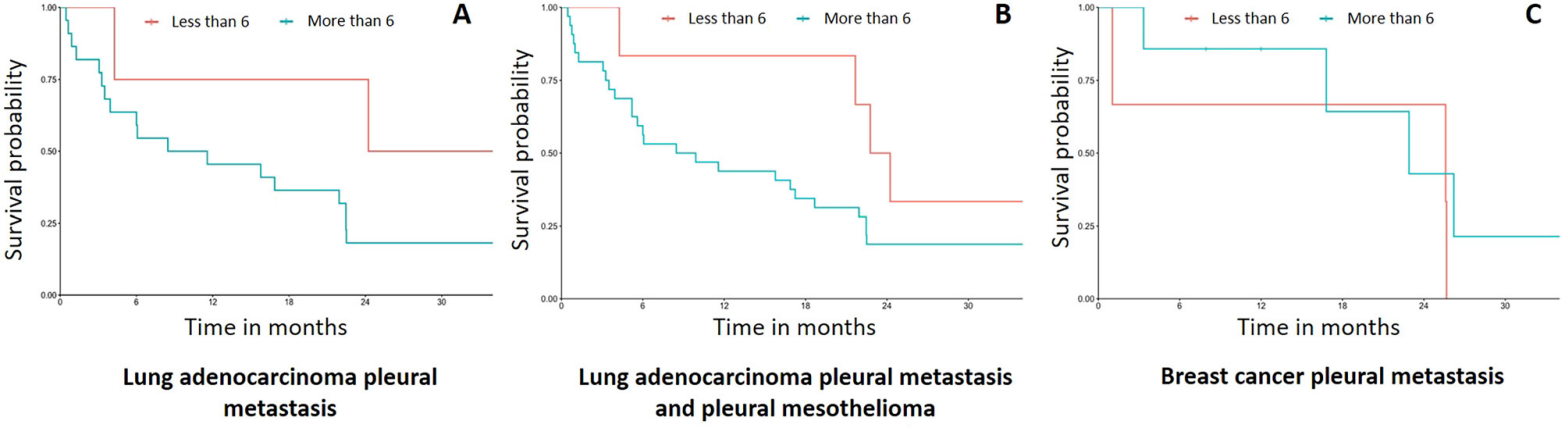
Overall survival of patients with lung cancer pleural metastasis according to their score.

Survival according to the pleural score in patients with pleural metastases from lung adenocarcinoma (LUADPM), breast cancer (BCPM) and mesothelioma (MPM) (most common aetiologies) has been studied. Patients with LUADPM who had a score of 6 or less tended to have a better survival than those with a higher score (Figure 3). This trend was maintained when LUADPM patients were grouped with MPM patients. On the other hand, survival of patients with BCPM was the same regardless of their score.

## Discussion

Malignant pleural effusion continues to be considered as a terminal oncologic event due to its poor prognosis [11] and the surgical strategy remains palliative [12], [13], [14]. From what has been done in peritoneal malignancies, we aimed to develop an easy-to-use, standardized scoring system for pleural carcinomatosis. Our study describes an accurate pleural score (AUC=93.0) that could help selecting patients for locoregional therapies.

For patients with no adhesions in the pleural space, we explored the pleura meticulously and carried out the recommended treatment, i.e. pleurodesis with talc, even if 34 % of patients were considered resectable by the referring surgeon. In the past, a similar situation was observed in patients with peritoneal metastasis. The limited effectiveness of systemic treatments led to the development of locoregional approaches such as hyperthermic intraperitoneal chemotherapy (HIPEC) [10] and, in unresectable cases, pressurized intraperitoneal aerosol chemotherapy (PIPAC) [15]. In the same way, new therapeutic local approaches in pleural carcinomatosis are needed. Hyperthermic intrathoracic chemotherapy (HITHOC) combined with pleurectomy has shown to be safe and provided prolonged disease-free and overall survival in selected patients with malignant pleural mesothelioma and pleural recurrences of thymoma [16], [17], [18]. HITHOC has sporadically been performed in lung [19], 20], ovarian [21], [22], [23] and breast cancer [24]. A meta-analysis, gathering several indications confirmed that HITHOC combined with cytoreduction surgery (CRS), regardless of primary malignancy, was safe and effective in controlling pleural effusion and increased survival rate in selected patients [25]. However, the major flaw is that there is presently no standardized pleural score, such as the Peritoneal Cancer Index (PCI) for peritoneal malignancies, to determine which patients would benefit most from this type of treatment.

We build in a score allowing reproducible analysis of the pleura that could help selecting patients for surgery. We propose, based on the present study and regardless of general condition, medical history, and possible other metastatic sites, that a score of 6 or lower indicates potential eligibility for curative intent surgery (CRS and HITHOC) and should lead to a discussion in a multidisciplinary tumor board. Scores between 6 and 9 fall into a grey area where surgical resection should be considered on a case-by-case basis. A score above 10 appears to be prohibitive for surgical resection. If resection seems unreasonable, whatever the reason, pressurized intrathoracic aerosol chemotherapy (PITAC) can be proposed as an alternative option of talc pleurodesis [26], [27], [28], [29], [30], in expert centers. In our cohort, two patients were considered resectable and could have undergone aggressive surgical treatment. In hindsight, they were relatively young, with no significant medical history, presenting lung cancer metastatic to the pleura without other metastatic sites. But, this surgical therapeutic approach was not recommended at that time.

In previous studies, no significant correlation was found between survival and thoracoscopic lesion rating [31], 32]. Only the KPS score at the time of thoracoscopy and adhesions in the pleural space seemed to be predictive of survival [33], 34]. Our study also failed to establish a significant relationship between score and survival, although patients with LUADPM with a score less than or equal to 6 appeared to have better survival. On the contrary, survival for patients with BCPM did not seem correlated with the pleural score. This is key information that could help refine the indications for surgery in pleural metastasis as it has been done in peritoneal disease [15]. Our visual assessment score could complement existing non-surgical scores such as the LENT [35] and the PROMISE [36] scores. Altogether, these scores should allow clinicians to better identify patients and tailor their care, avoiding over-invasive treatments in fragile end-stage patients and under-treatment in patients who are in good shape or in the “early” stage of their disease. This pleural score could be completed afterwards by pathological, radiological and molecular data to enhance its accuracy. In peritoneal cancers, radiological and pathological scores have been developed (rPCI and pPCI) [37] as there is some correlation between serosal thickness on CT scan images and survival, and because pathologic analyses allow a precise tumor burden estimation. Additionally, the pleural score could incorporate biomarkers and molecular profiling to enhance its prognostic capabilities and guide local or systemic targeted therapies. If widely used, before and/or after therapies, a multimodal pleural score could facilitate comparison of treatments outcomes across institutions and clinical trials, accelerating the identification of optimal management of pleural metastasis.

However, this study has some limitations. Due to the small number of patients, which we attempted to compensate for with a bootstrap validation, our score requires further validation in prospective and larger-scale studies. A study with a larger number of patients could also involve subgroup analyses, study the score according to the primary tumor, as it is considered the most important factor influencing survival [3], and make this score even more accurate. Our next step is to validate the proposed score prospectively, and therefore to operate on patients with a score of 6 or less, and to assess if complete resection can be obtained, and if survival is longer compared to medical treatment alone.

## Conclusions

A standardized pleural scoring system could improve pleural metastasis management. This easy-to-use score based on per operative observations could help select patients for locoregional therapies. Patients with a score equal to or less than 6 should be discussed in a multidisciplinary tumor board where surgery is considered. Patients with a score greater than 6 should be discussed for less invasive management such as PITAC procedures or talc pleurodesis. The steps forward are now to test the score on larger scale studies to validate its accuracy, to evaluate the oncological benefits of surgery in this context and to upgrade the score by incorporating radiological, pathological and molecular data.
